# Barriers to credentialing emergency physicians in ultrasound use

**DOI:** 10.1186/2036-7902-4-S1-A11

**Published:** 2012-12-18

**Authors:** Bret P Nelson, J Mahesri, A Huang

**Affiliations:** 1 Department of Emergency Medicine, Mount Sinai School of Medicine, New York, USA

## Background

Ultrasound training and hospital credentialing guidelines were established by the American College of Emergency Physicians (ACEP) in 2001. Training during residency has become the norm yet the penetration of hospital credentialing for practicing emergency physicians is unknown.

## Objective

To investigate the availability of emergency ultrasound credentialing and what barriers to credentialing exist.

## Patients and methods

An online survey was distributed through the ACEP ultrasound section. It consisted of questions regarding credentialing pathways, ultrasound use, and barriers to credentialing.

## Results

Of 195 respondents, 85% were board certified in emergency medicine with a mean age of 40 years. 69% practiced in academic hospitals, 27% in community, and 4% in military. 83% worked in departments with annual volumes >40,000 visits and 92% had an emergency ultrasound director. Credentialing mechanisms existed for 96% of respondents; 51% of hospitals used ACEP guidelines for credentialing. Credentialed respondents were credentialed in: FAST (78%), Vascular (74%), Aorta (68%), OB/Gyn (66%), Gallbladder (55%), Renal (53%), and DVT (40%). Non-credentialed respondents most commonly cited “lack of experience” (35%) and “too busy” (29%) as barriers. Academic and community physicians were credentialed at the same rate. Those who completed training prior to 2001 were less likely to be credentialed than those trained after in all areas except gallbladder ultrasound. Financial incentive (34%) and hands on experience (31%) were most often cited as reasons to pursue credentialing. This was true for those trained before or after 2001, and for academic or community practice.

## Conclusion

While most surveyed centers allow for credentialing, many physicians are still not credentialed. Based on these results, targeting physicians trained prior to 2001 with financial incentives and opportunities for hands-on ultrasound experience may be of benefit.
